# Functional Characteristics of the Gut Microbiome in C57BL/6 Mice Differentially Susceptible to *Plasmodium yoelii*

**DOI:** 10.3389/fmicb.2016.01520

**Published:** 2016-09-27

**Authors:** Joshua M. A. Stough, Stephen P. Dearth, Joshua E. Denny, Gary R. LeCleir, Nathan W. Schmidt, Shawn R. Campagna, Steven W. Wilhelm

**Affiliations:** ^1^Department of Microbiology, University of TennesseeKnoxville, TN, USA; ^2^Department of Chemistry, University of TennesseeKnoxville, TN, USA; ^3^Department of Microbiology and Immunology, University of LouisvilleLouisville, KY, USA

**Keywords:** microbiome, C57BL/6N lineage, metatranscriptome, malaria, metabolome

## Abstract

C57BL/6 mice are widely used for *in vivo* studies of immune function and metabolism in mammals. In a previous study, it was observed that when C57BL/6 mice purchased from different vendors were infected with *Plasmodium yoelii*, a causative agent of murine malaria, they exhibited both differential immune responses and significantly different parasite burdens: these patterns were reproducible when gut contents were transplanted into gnotobiotic mice. To gain insight into the mechanism of resistance, we removed whole ceca from mice purchased from two vendors, Taconic Biosciences (low parasitemia) and Charles River Laboratories (high parasitemia), to determine the combined host and microflora metabolome and metatranscriptome. With the exception of two Charles River samples, we observed ≥90% similarity in overall bacterial gene expression within vendors and ≤80% similarity between vendors. In total 33 bacterial genes were differentially expressed in Charles River mice (*p*-value < 0.05) relative to the mice purchased from Taconic. Included among these, *fliC*, *ureABC*, and six members of the *nuo* gene family were overrepresented in microbiomes susceptible to more severe malaria. Moreover, 38 mouse genes were differentially expressed in these purported genetically identical mice. Differentially expressed genes included *basigin*, a cell surface receptor required for *P. falciparum* invasion of red blood cells. Differences in metabolite pools were detected, though their relevance to malaria infection, microbial community activity, or host response is not yet understood. Our data have provided new targets that may connect gut microbial activity to malaria resistance and susceptibility phenotypes in the C57BL/6 model organism.

## Introduction

Since its development in the 1940’s, the C57BL/6 inbred mouse strain has become one of the most widely used murine genetic backgrounds for diverse biomedical research. The strength of these inbred mice as model organisms is their reproducibility, allowing independent researchers to carry out experiments on genetically identical mice (Silver, 1995). Use of this inbred strain became so widespread it was selected as the first murine genome to be sequenced (Mouse Genome Sequencing Consortium et al., 2002). However, in recent years, attention has been drawn to the split in the strain’s ancestral line during the 1950’s when mice were separately bred and maintained by the National Institutes of Health (NIH) and Jackson Laboratory, now known as C57BL/6N and C57BL/6J, respectively (Bailey, 1978; Altman and Kats, 1979). Concern has arisen over use of these divergent substrains interchangeably as model organisms following multiple reports of changes in behavior (Crawley et al., 1997), differential tolerance to ethanol (Khisti et al., 2006; Green et al., 2007), deletion of the gene encoding nicotinamide nucleotide transhydrogenase (*nnt*) in the C57BL/6J lineage (Freeman et al., 2006), and discovery of multiple SNPs between derived mouse genomes (Mekada et al., 2009). The importance of these strains to the scientific community has led to major efforts to describe the genomic (Simon et al., 2013) and regulatory (Keane et al., 2011) differences between the various lineages, and catalog them for proper selection of model organisms (Grubb et al., 2014).

While the genetic differences and the resulting phenotypic alterations between the major C57BL/6 lineages may be increasingly considered by researchers during experimental design, only recently can this be said for their “second genome”: the microbiome. The importance of tissue-associated microbial symbionts to mammalian metabolism and immunity has become well established. Gut microbial communities in particular make up the majority of the microbial consortia and diversity in the body (Savage, 2002), and play an important role in early post-natal development of the immune system, protection from gut pathogens, and host metabolism. Members of the taxa Firmicutes and Bacteroidetes dominate intestinal communities, largely responsible for the catabolism of hundreds of different glycans indigestible by mammalian enzymes, giving the host access to otherwise recalcitrant nutrients (Backhed et al., 2005). The resulting pool of monosaccharides are fermented to short-chain fatty acids, which not only provide energy for the host, but have been shown to influence immune function. Acetate and butyrate, influenced by dietary fiber content, can signal through G-protein-coupled receptors expressed on CD4+ T helper cells resulting in the regulation of cytokine expression and resolution of intestinal inflammation (Kau et al., 2011). Indeed, just as immune cells use receptors to detect infection and tissue damage signals, it is apparent that the same receptors are used in different combinations to detect beneficial microbial activity and prevent harmful response (Swiatczak and Cohen, 2015). Despite the profound influence that even subtle changes in gut community composition and activity can have on host physiology, the impact of the gut microbiome on mice used as model organisms remains poorly understood.

It was recently shown that when C57BL/6 mice purchased from different vendors were infected with malaria parasite *Plasmodium yoelii*, they exhibited significantly different parasite burdens and immune responses. This was confirmed to be the result of microbial interaction with the mouse host when both resistant and susceptible phenotypes were reproduced via fecal transplant to gnotobiotic mice (Villarino et al., 2016). Subsequent sequencing of 16S rRNA gene libraries obtained from the transplanted gut microbiomes showed conservation in gut microbial community composition within, but major differences between, samples obtained from mice from different vendors. To elucidate the mechanisms underlying microbiome-mediated resistance to malaria, the cecum microbial and host metatranscriptome was sequenced. Significant differences were observed in both host and bacterial transcription patterns. Additionally, the metabolic profiles of cecum whole tissue samples were determined and analyzed. Overall differences in individual metabolite concentrations call into question the interchangeable use of mice from different sources. These data begin to elucidate factors that may influence susceptibility to *P. yoelli* infection, and these results also provide further evidence that caution is needed when comparing results from experiments using mice from separate C57BL/6 sublineages and/or vendors.

## Materials and Methods

### Mice and Infections

Female C57BL/6 mice were purchased from Taconic Biosciences (Hudson, NY, USA) and Charles River Laboratories (Wilmington, MA, USA). Mice were housed and maintained at University of Tennessee animal care facility under biosafety level 2 conditions. Mice were fed NIH-31 Modified Open Formula Mouse/Rat Irradiated Diet (Envigo 7913; Envigo, Indianapolis, IN, USA) and provided autoclaved municipal tap water to drink. To verify vendor-dependent malaria disease severity, mice were infected with 10^5^ *P. yoelii* parasitized red blood cells (pRBCs) *via* tail vein injection after a 2 week acclimation period upon arrival at the animal care facility. Parasite burden was determined from thin blood smears. Blood samples were obtained by performing tail snips. Slides were fixed with methanol, followed by Giemsa stain (Thermo Fisher Scientific) diluted 1:20 in ddH_2_0 for 30 min. Percent parasitemia was calculated as the percent of total RBCs that contain a blood stage parasite averaged from the counts RBCs within a 10 × 10 grid from five microscope fields (1000x) per sample. All studies were performed in accordance with the recommendations in the Guide for the Care and Use of Laboratory Animals of the National Institutes of Health and approved by the University of Tennessee Institutional Animal Care and Use Committee.

### Gut Microbiome Sampling

As sampling directly from gut tissue is destructive, mice used for microbiome sampling were not used to track parasite burden. To limit potential variation in gut microbial communities and to ensure that the disease severity phenotype was consistent, mice used for microbiome sampling were purchased in the same batch as those used for tracking disease progression. Six mice from each vendor (Taconic Biosciences and Charles River Laboratories) were acclimated for 2 weeks upon arriving at the animal facility. After acclimation the mice were sacrificed and a necropsy performed. Whole ceca were removed, weighed, and immediately flash frozen in liquid nitrogen and stored at -80°C. Cecum samples were divided in half for metabolomics analysis and metatranscriptome sequencing.

### RNA Extraction and Sequencing

Total RNA was isolated from whole ceca using the MOBIO Power Microbiome^TM^ RNA extraction kit. RNA concentration and purity was determined using a NanoDrop ND-1000 spectrophotometer. Measurements were taken three times to account for variability in the readings. Extracted RNA was tested for DNA contamination by running a polymerase chain reaction using universal bacterial 16S rRNA primers 27F and 1492R. DNA contamination was removed with the MOBIO RTS DNase kit. Twelve purified RNA samples were shipped to the Hudson Alpha Institute Genomic Services Laboratory (Huntsville, AL, USA) for rRNA reduction and sequencing on the Illumina HiSeq platform using a paired-end 100bp flow cell.

### Metabolite Extraction and Analysis

Contents were removed from ceca placed in 1.5 mL centrifuge tubes and suspended in 1.3 mL of extraction solvent (40:40:20 HPLC grade methanol, acetonitrile, water with 0.1% formic acid) kept at 4°C. Extraction proceeded for 20 min at -20°C before samples were centrifuged for 5 min (16.1 rcf) at 4°C and supernatants were transferred to new vials. The remaining cecal contents were resuspended in 200 μL of cold (4°C) extraction solvent. The extraction was again allowed to proceed for 20 min at -20°C before being centrifuged for 5 min (16.1 rcf) at 4°C. These supernatants were also transferred to the vials and another 200 μL of extraction solvent was added to the pelleted cell for a final wash by repeating the previous extraction once more. The vials containing all of the combined extraction supernatants were placed in a nitrogen drying apparatus until all the extraction solvent had been evaporated. The residual solid was resuspended in 300 μL of sterile water and transferred to 300 μL autosampler vials. Samples were immediately placed in a 4°C autosampler for mass spectrometric analysis.

A 10 μL injection of each sample was separated through a Synergi 2.5 micron Hydro-RP 100 Å, 100mm × 2.00 mm LC column (Phenomenex, Torrance, CA, USA) maintained at 25°C. The mass spectrometer and chromatographic separation were performed similar to a reported method (Lu et al., 2010). The eluent was introduced into the mass spectrometer *via* an electrospray ionization source in negative mode before entering an Exactive Plus orbitrap mass spectrometer (Thermo Scientific, Waltham, MA, USA) through a 0.1-mm internal diameter fused silica capillary tube. The samples were run with a spray voltage of 3 kV, a nitrogen sheath gas flow rate of 10 units, a capillary temperature set at 320°C, and an AGC target set to 3e6. The samples were analyzed in full scan mode with a resolution of 140,000 and a scan window of 85 to 800 *m/z* for from 0 to 9 min and 110 to 1000 *m/z* from 9 to 25 min. Solvent A consisted of 97:3 HPLC grade water:methanol, 10 mM tributylamine, and 15 mM acetic acid. Solvent B was HPLC grade methanol. The mobile phase gradient from 0 to 5 min was 0% B, from 5 to 13 min was 20% B, from 13 to 15.5 min was 55% B, from 15.5 to 19 min is 95% B, and from 19 to 25 min was 0% B while maintaining a constant flow rate of of 200 μL/min.

### Data Processing

Raw sequences were downloaded from the HudsonAlpha Institute server and checked for quality using FastQC application (Babraham Institute, Cambridge, England). Unless noted, all bioinformatics and statistical software were used at default settings. Samples were subjected to a subsequent *in silico* rRNA reduction using the SortmeRNA 2.0 software package (Kopylova et al., 2012). Since RNA was extracted from whole cecum tissue and would contain mRNA of murine origin, processed reads were paired and mapped to the *Mus musculus* reference genome using the CLC Genomics Workbench v8.5 (Waltham, MA, USA). Mouse reads were annotated and further analyzed in CLC. Unmapped reads were assumed to originate from the gut microbiome and were uploaded to the Metagenomics RAST server (MG-RAST; Meyer et al., 2008) for alignment and identification. All sequencing data were submitted to the Short Reads Archive (SRA) under accession code SRP075802.

For metabolome data, raw files generated by Xcalibur were converted to the open-source mzML format (Martens et al., 2011) *via* the ProteoWizard package (Chambers et al., 2012). MAVEN software (Clasquin et al., 2012; Princeton University) was used to automatically perform non-linear retention time correction for each sample. Metabolites were manually identified by *m/z* (±5 ppm) and retention time for each sample using MAVEN to calculate associated peak areas. Relative concentrations (*i.e.*, in the absence of internal standards for all metabolites) were normalized by mass of the processed tissue sample. Fold changes were calculated and the data were transformed and clustered using Cluster software (de Hoon et al., 2004). Heat maps were generated from clustered data using Microsoft Excel software.

### Statistical Analysis

Microbial transcript abundances annotated from the SEED Subsystem database (Overbeek et al., 2005; evaluated as raw read counts) were exported from the MG-RAST server and normalized by library size. Normalized gene expression data and relative metabolite concentration were log transformed, and used to generate a Bray-Curtis dissimilarity matrix and non-metric multidimensional scaling plots in the PRIMER7 software suite (Clark and Gorley, 2015). PRIMER7 was also used to perform ANOSIM tests comparing overall expression and metabolite profiles. Differences in individual gene expression, between gut microbial communities from the two vendors, were determined using the edgeR Bioconductor package in R Statistics software (Robinson and Smyth, 2007, 2008; Robinson et al., 2010; McCarthy et al., 2012; Zhou et al., 2014). Differential expression of individual mouse genes between vendors was determined using the edgeR test implemented in CLC Genomics Workbench. Figures were generated using SigmaPlot (Systat Software, Inc.). As *p*-values from statistical tests were false discovery rate adjusted for multiple comparisons, a *p*-value cutoff of 0.1 was used to provide thorough detailing of differences between mouse substrains that may be useful to researchers. Additionally, Cohen’s *d* effect size (Cohen, 1988) was calculated for each gene from relative transcript abundances. All significantly different genes and metabolites are presented with their *p*-values, fold changes, and effect sizes in the Supplementary Material.

## Results

### Differential Susceptibility to *P. yoelii*

**FIGURE 1:**
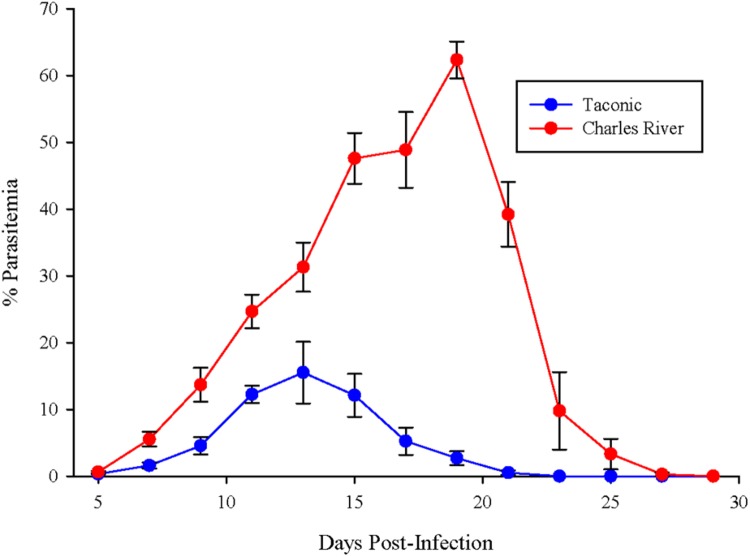
**C57BL/6 mice from Taconic exhibit reduced parasitemia compared to mice from Charles River.** Mice were infected with 10^5^ *P. yoelii* pRBCs. Percent parasitemia was determined on the indicated days. Data (mean ± SD) are cumulative results (*n* = 7–8 mice per group) from two independent experiments.

C57BL/6N mice from Taconic and Charles River were infected with *P. yoelii* pRBCs. Parasitemia in Taconic mice peaked 13 days post-infection at ∼15% and was cleared by 23 days post-infection (**Figure 1**). Charles River mice exhibited higher parasite burden, peaking at ∼60% parasitemia 19 days post-infection and delayed clearance (day 29 post-infection) compared to Taconic mice (**Figure 1**). These data are consistent with previous observations that showed *P. yoelii* infection of C57BL/6 mice from Taconic and Jackson Laboratories had lower parasitemia than C57BL/6 mice from Charles River, National Cancer Institute, and Envigo (formally Harlan; Villarino et al., 2016).

### Transcriptome Results

Ribosomal RNA reduction, cDNA synthesis, and sequencing on the Illumina HiSeq yielded a total of 294 million paired-end 100bp reads across 12 samples. An average of 43.8% of reads were removed during *in silico* rRNA reduction using SortMeRNA. One of the Taconic samples exhibited much higher attrition, with 73.2% of its reads removed. As a result, the number of reads annotated from this sample were a full order of magnitude lower than the other samples, so it was removed from further analyses because of dissimilarity. Reads passing quality control were mapped to the mouse genome and subsequently used to determine murine transcriptional patterns. The remaining reads were uploaded to MG-RAST for characterization of microbial transcriptional patterns. The quality control pipeline removed an average of 14.6% of reads due to read quality, artificial duplication, and estimated sequencing error. An average of 2.1 million reads per sample were annotated as microbial transcripts and divided into functional categories.

### Community Structure and Function

**FIGURE 2:**
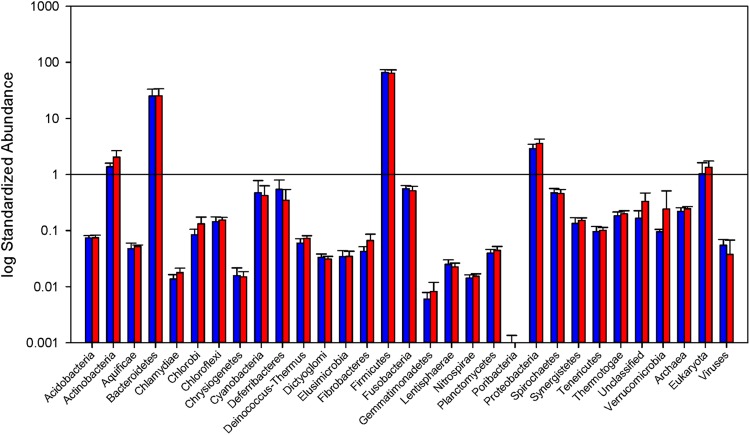
**Relative abundance of Bacterial phyla and total Archaea, Eukarya, and virus reads.** Read counts normalized by library size from the samples in each group. Blue bars represent abundance in mice purchased from Taconic Biosciences. Red bars represent abundance in mice purchased from Charles River Laboratories. Error bars represent standard deviation. Data (mean ± SD) are from *n* = 5 Tac and *n* = 6 CR mice.

The phylogenetic makeup of the cecal microbial community as determined by metatranscriptomic analysis is represented in **Figure 2.** The microbial community transcriptional profile is dominated by the bacterial phyla Firmicutes and Bacteroidetes, the reads from which make up an average of 90.1% ± 6.3 of each sample. The next most abundant source of transcripts originate in Proteobacteria at 3.2% ± 0.20 of reads, followed by Actinobacteria, 1.7% ± 0.16, and Fusobacteria, 0.53% ± 0.03. Within the phylum Bacteroidetes, families Bacteroidaceae and Porphyromonadaceae are most prevalent, 46.7% and 51.1% of the phylum, respectively. The Firmicutes portion of the community is split predominantly between orders Lactobacillales and Clostridiales, 8.9 and 82.5% of the phylum, respectively. The MG-RAST pipeline identified 0.04% ± 0.007 of the reads as being of viral origin, all of which were bacteriophage. Archaea made up 0.24% ± 0.009 of the transcripts, with the Euryarchaeota dominating at 92.3% of the Archaeal reads.

**FIGURE 3:**
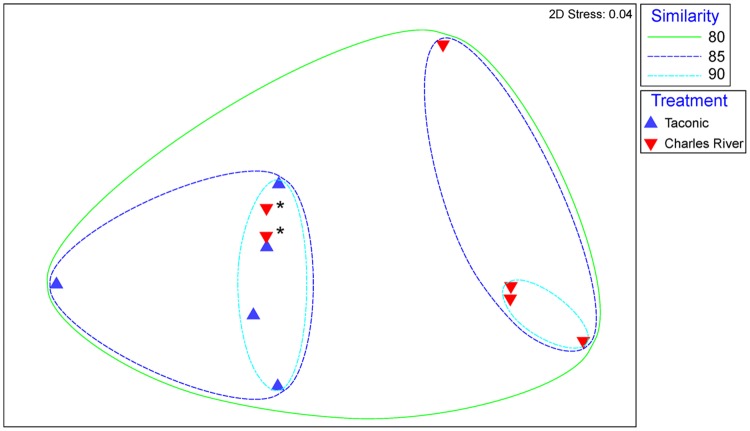
**Non-metric multidimensional scaling of Bray-Curtis similarity matrix comparing overall abundances of bacterially derived transcripts.** Blue points represent samples isolated from Taconic Biosciences mice. Red points represent samples isolated from Charles River Laboratories mice. Ellipses represent lines of 80, 85, and 90% similarity between samples. Asterisks designate two Charles River samples addressed in text.

Non-metric multidimensional scaling plot of Bray–Curtis dissimilarity analysis is represented in **Figure 3.** Sample Taconic 6 was left out of this analysis due to significant dissimilarity caused by methodology that skews the plot. Overall bacterial transcript abundances in the 5 Taconic and 6 Charles River samples are at least 80% similar **Figure 3.** With the exception of two Charles River samples (designated by asterisks in **Figure 3**), mouse groups cluster with at least 85% similarity and as high as 98%. These two samples more closely resemble expression profiles of the Taconic gut communities. As mice from these two substrains are so closely related, some overlap within the internal variation of the mouse groups was to be expected. However, ANOSIM analysis comparing overall expression of bacterially derived transcripts determined that community expression between mouse groups was statistically different (*p* = 0.048).

**FIGURE 4:**
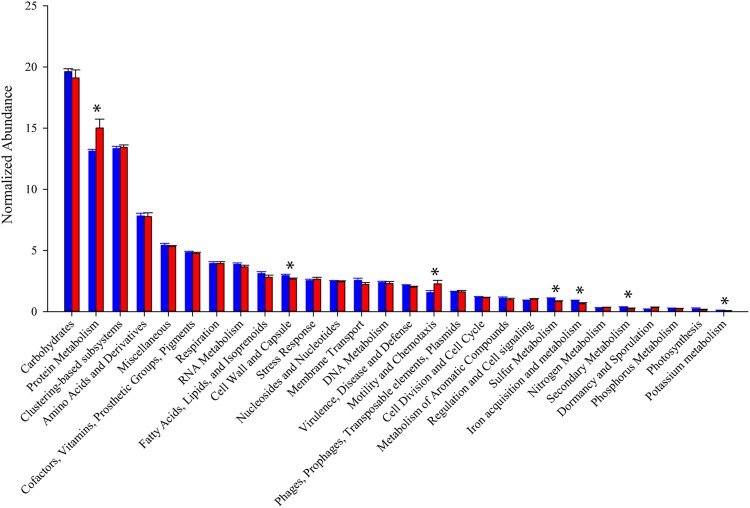
**Relative abundance of SEED subsystems functional categories.** Read counts normalized by library size from samples within each group. Blue bars represent abundance in mice purchased from Taconic Biosciences. Red bars represent abundance in mice purchased from Charles River Laboratories. Data (mean ± SD) are from *n* = 5 Tac and *n* = 6 CR mice. Asterisks indicate functional categories significantly different (*p* < 0.05) or trending toward significant (*p* < 0.8) in a comparison via unpaired Student’s *t*-test.

In general, the distribution of sequences within SEED Subsystem categories were consistent between the two mouse groups (**Figure 4**). Combining 11 metatranscriptomes, the most abundant functional groups are Carbohydrate Metabolism (19.5%), Protein Metabolism (14.0%), and Amino Acid Metabolism (7.7%). A significant portion (13.3%) of the sequences are categorized as clustering-based subsystems, whose functions are bioinformatically identified, but not yet experimentally validated. An unpaired *t*-test comparing normalized expression of individual Level 1 SEED Subsystem categories between the two treatment groups yielded significant (*p* < 0.05), or trending toward significant (*p* < 0.08), differences in Protein Metabolism (*p* = 0.029), Cell Wall and Capsule synthesis (*p* = 0.053), Motility and Chemotaxis (*p* = 0.047), Sulfur Metabolism (*p* = 0.038), Iron Acquisition and Metabolism (*p* = 0.077), Secondary Metabolism (*p* = 0.059), and Potassium Metabolism (*p* = 0.014).

### Differentially Expressed Bacterial Genes

**FIGURE 5:**
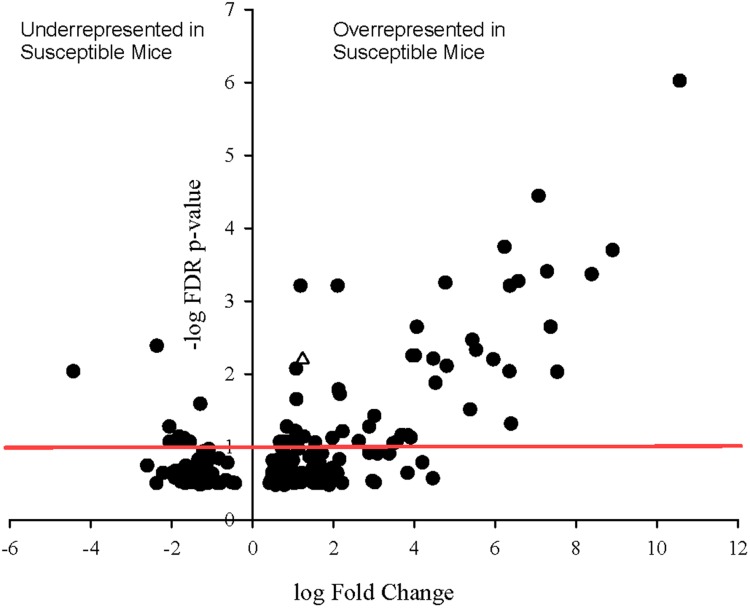
**Volcano plot showing degree of differential expression of bacterially derived genes in Charles River Laboratories mice compared to Taconic Biosciences.** Log-transformed fold change in expression is plotted on the x-axis and log-transformed false discovery rate-adjusted *p*-values plotted on the y-axis. The red horizontal line represents the 0.1 *p*-value cutoff. Empty triangle: *fliC* (Flagellin).

To determine whether specific transcripts significantly differed in expression between the resistant and susceptible phenotypes, statistical analysis of differential gene expression of bacterially derived transcripts was performed using the edgeR Bioconductor package. A total of 60 bacterial genes were differentially expressed (*p* ≤ 0.1), 33 of which with false discovery rate (FDR) adjusted *p*-values less than 0.05 and 11 with *p*-values less than 0.001 (**Figure 5**). Of these, 51 of 60 genes were overrepresented in Charles River mice compared to Taconic. The majority of differentially expressed genes are involved in energy, amino acid, and carbon metabolisms. Overexpressed in Charles River mice were transcripts encoding FliC, the flagellar body protein, which is heavily proinflammatory. Only three genes were determined to be significantly overrepresented in resistant mice purchased from Taconic Biosciences. All statistically significant bacterial genes, with the exception of three, exhibited an effect size greater than 0.8, the value typically used as the cutoff for a strong effect.

### Differentially Expressed Mouse Genes

**FIGURE 6:**
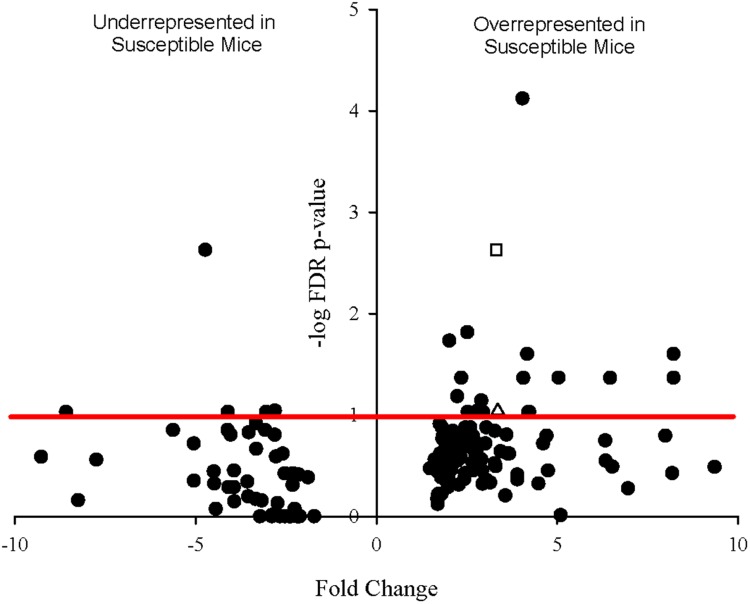
**Volcano plot showing degree of differential expression of mouse-derived genes in Charles River Laboratories mice compared to Taconic Biosciences.** Log-transformed fold change in expression is plotted on the x-axis and log-transformed false discovery rate-adjusted *p*-values plotted on the y-axis. The red horizontal line represents the 0.1 *p*-value cutoff. Empty square: *bsg* (Basigin). Empty triangle: *lgals9* (Galectin-9).

Since sequencing also yielded mouse transcripts within the samples, differential gene expression amongst the murine transcripts was also analyzed. Fold change in gene expression and FDR adjusted *p*-values from the exact test are presented in the volcano plot in **Figure 6.** Twenty genes were differentially expressed with a *p*-value less than 0.1, 12 of which had *p*-values less than 0.05. Of these, 11 genes were significantly overrepresented in Charles River mice and one in Taconic mice. The overrepresented transcripts in Charles River mice include Galectin-9 (*LGALS9*), which is an important immune signaling molecule (Merani et al., 2015), and Basigin (*bsg*), a cell surface receptor whose expression is required for infection of RBCs by the human malaria parasite *P. falciparum* (Crosnier et al., 2011). All statistically significant mouse genes exhibited an effect size greater than 1.0, with the lowest being 1.16.

### Metabolite Pools

**FIGURE 7:**
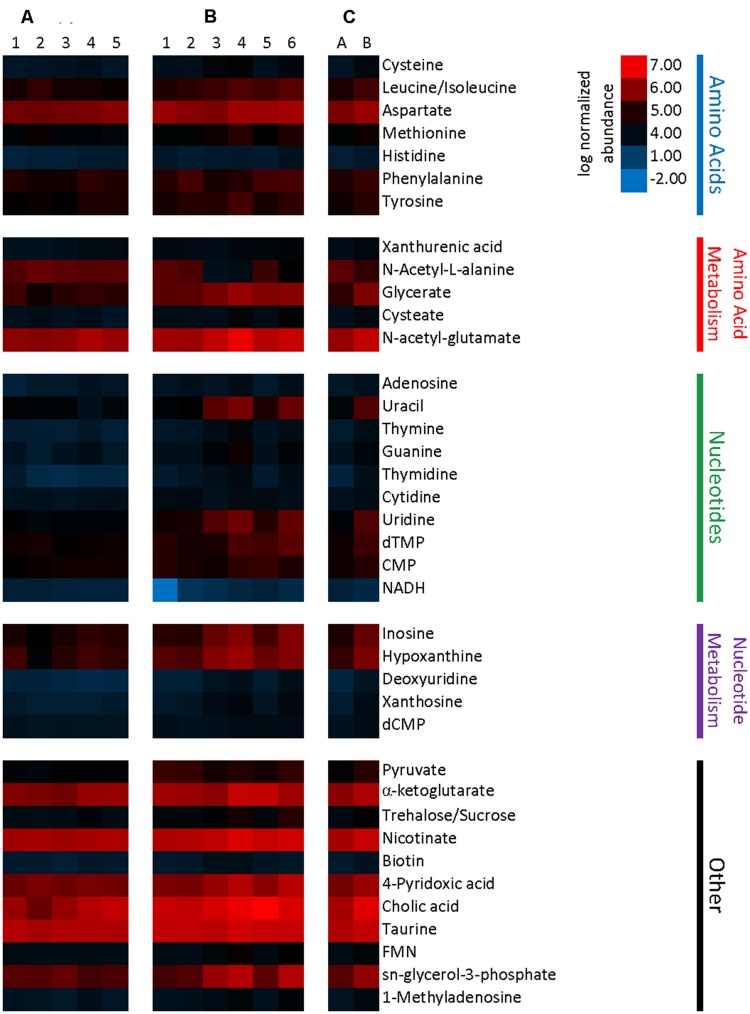
**Heatmap representing metabolite abundances normalized to sample tissue mass and log transformed.** Metabolites displayed are significantly different with a *p*-value cutoff of 0.1. **(A)** Five columns represent metabolite abundances for each of five Taconic Biosciences mice. **(B)** Six columns represent each of the six Charles River mice. **(C)** Columns represent the mean abundances for Taconic **(A)** and Charles River **(B)**.

Relative metabolite concentrations were normalized by mass of the processed tissue sample, and these data were used to calculate fold change and cluster analyses. Comparison of normalized metabolite abundances determined that differences in the metabolome of Charles River and Taconic mice were present (*p* = 0.082). Normalized abundance of significantly different metabolites are presented in **Figure 7.** Of the 129 metabolites detected in the samples, 36 were found in significantly higher relative concentrations in Charles River mice, and two (NADH and N-acetyl-L-alanine) were found in higher concentrations in Taconic mice (*p* < 0.1). All statistically significant metabolites exhibited an effect size greater than 1.0, with the lowest being 1.17. The majority of significant metabolites were nucleotides, amino acids, or the substrates involved in the biosynthesis of these compounds. While a number of additional transcripts and metabolites were differentially abundant between mouse substrains, we have restricted our discussion to only those where a mechanism influential in gut microbial symbiosis, immune regulation, and malaria infection are clear.

## Discussion

Previous studies have demonstrated that of the microbiome of C57BL/6 mice can modulate the severity of *Plasmodium* infections in mice (Villarino et al., 2016). The resistant and susceptible phenotypes were not only reproducible across cohorts, but transmissible as part of cecal transplants to germ-free mice. Differences in parasite burden and bacterial community composition of Taconic and Charles River mice in the current study were consistent with previous research. Taconic mice exhibited significantly lower peak parasite burden and recovered from infection more quickly than Charles River mice. These findings strongly suggest that, as with our previous study, differences in parasite burden are the result of some currently unidentified interaction between the host and the gut microbiota, rather than the effects of epigenetic regulation, genetic or environmental effects. However, differential expression of mouse genes and differential abundance of metabolite pools are purely associative until further gut transplant studies are carried out.

Phylogenetically, the vast majority of transcripts were produced by bacteria, with Bacteroidetes and Firmicutes the most abundant among them. And while reliance on transcript abundance as an indicator of community composition is tenuous, the data are consistent with 16S rRNA and metagenomic studies of both mice and humans (Backhed et al., 2005; Ley et al., 2006; Sekirov et al., 2010). Overall, community composition inferred from transcript abundance did not differ at the phylum level between mice from the two vendors sampled. However, relevant differences in community functional profiles from overall expression patterns suggest that the factors involved in affecting host phenotype may exist at a finer scale.

Within the context of our study neither Archaea nor viruses make up a significant portion of transcriptional activity, although their contribution cannot be discounted. Previous studies have also shown their abundance is lower than their bacterial counterparts (Hoffmann et al., 2013); however, it is likely that this community was not sequenced deeply enough to detail their role. Viruses in particular may require targeted approaches to better resolve their influence on community dynamics and host phenotype. The role of phage populations may be limited to top-down control of the bacterial community with no direct influence over host cells (Ogilvie and Jones, 2015).

Differential bacterial gene expression in the cecum, in part, reflects differences in microbial community composition between mouse strains that are often used interchangeably in research and provides important targets to unveil the mechanism underlying resistance to malaria. Overrepresentation of transcripts encoding flagellin in Charles River mice suggests a mechanism that may involve indirect modulation of the immune system by the gut microflora. Flagellin is the principal protein component of the bacterial flagellum, encoded by the gene *fliC*. While the majority of the gut microbial diversity is capable of producing flagella, flagellin levels are generally low in the healthy gastrointestinal tract (Verberkmoes et al., 2009). Increased flagellin expression can be associated with mucosal barrier breakdown and inflammation (Sanders, 2005; Gewirtz, 2006). It has been hypothesized that anti-flagellin antibodies down-regulate *fliC* expression in resident non-pathogenic microbes (Cullender et al., 2013) and this prevents colonization by potential pathogens (Ghose et al., 2016). However, it is currently unclear whether local stimulation of innate and adaptive immune response in the gut *via* Toll-like receptor 5 (TLR5; Gewirtz et al., 2006) is relevant to the immune response to *Plasmodium* infection.

Differential regulation of murine gene expression between groups of mice purchased from different vendors is compelling evidence of non-genomic C57BL/6N strain divergence. Of particular interest is the overrepresentation of basigin (BSG) in Charles River mice and its possible involvement in malaria resistance. Also referred to as CD147 or EMMPRIN (extracellular matrix metalloprotease inducer), basigin is a cell surface receptor in the immunoglobulin superfamily. It is commonly expressed on many tissue types and is involved in a wide variety of biological functions, such developmental processes, nutrient transport, and inflammation (Xiong et al., 2014; Hahn et al., 2015). The basigin gene, *bsg*, can encode four different variants through alternative splicing, each of which is expressed in different tissues (Liao et al., 2011). Subsequent assembly and analysis of Basigin transcripts from our dataset identified that the vast majority of reads encoded isoform Bsg-2, the most abundant and best characterized isoform in human and mouse tissue. While basigin is involved in many processes, it became relevant to human health when it was found to induce expression of matrix metalloproteases, which can promote tumor cell development, invasion, and metastasis (Hahn et al., 2015). Perhaps more relevant to the current work, a recent study identified Bsg-2 as a key receptor for reticulocyte-binding protein homolog 5 (PfRh5), the parasite ligand required for erythrocyte invasion by *P. falciparum* (Crosnier et al., 2011). In total these observations results in the new hypothesis that decreased expression of basigin isoform Bsg-2 in Taconic mice may contribute to their malaria resistance.

Another overrepresented transcript in Charles River mice encodes the β-galactoside-binding protein galectin-9. Galectins bind specifically to glycosylated proteins and are typically involved in cell signaling and regulation. As a result, dysfunction of galectin activity and expression is closely linked to cancer development (Thijssen et al., 2015) and autoimmune disorders (Blidner et al., 2015). As a ligand for the type-I glycoprotein Tim-3, galectin-9 modulates the innate immune response to viral infection by inducing apoptosis in infected T cells (Merani et al., 2015). Dysfunctional expression and activation of the Tim-3 signaling molecule has been linked to CD4^+^ and CD8^+^ T cell “exhaustion” in chronic HIV (Jones et al., 2008) and hepatitis C (Golden-Mason et al., 2009) infection. It is possible that underrepresentation of galectin-9 in Taconic mice may improve T cell response to *Plasmodium* infection. However, interest in galectin proteins as important immune signaling molecules has emerged only recently. As the regulation of these proteins is poorly understood, the mechanism by which the gut microbiota may influence galectin expression is unclear.

As part of our analysis we mapped both transcripts and metabolite data (*p* ≤ 0.1) onto microbial metabolic pathways to identify biological processes that may link the two. However, we were unable to find connections beyond two or three features within any pathway. This may be due to the relatively low transcript coverage of the vast metabolic capabilities of the microbiome, but is likely also related to the transient nature of gut contents and the constant flux of new material combined with the temporal disconnect between transcriptional and metabolic responses. Additionally, it can be difficult to determine whether differential relative concentrations of specific molecules are the cause or result of physiological change. However, the presence of significant differences in specific gut metabolites, as well as relevant difference in overall metabolite pools, between C57BL/6N mice is of serious concern to those that rely on them for reproducibility. Previous work has also shown that the murine microbiome can alter the concentration of circulating metabolite in the host (Villarino et al., 2016), further complicating the comparison of results between vendors and substrains.

This study identified key differences in the gene expression of both the microbial and murine components of the gastrointestinal tract, including the cell surface receptor basigin, as a potential link between the gut microbiome and the previously observed malaria resistance. Differential expression of the immune signaling protein galectin-9 was also noted, and this alteration may play a role in regulation of the differential immune response observed in the prior study. Additionally, a relevant difference in the overall metabolome and significant differences in multiple individual metabolites were observed. While the differences in gene expression and metabolism we observed provide evidence against the interchangeability of mice obtained from different vendors, they shed new light on potential avenues for investigation into the effects of the microbiome on the severity of malaria.

## Author Contributions

JS, SD, JD, GL completed the lab components and statistical analysis. JS lead the writing component with all authors contributing.

## Conflict of Interest Statement

The authors declare that the research was conducted in the absence of any commercial or financial relationships that could be construed as a potential conflict of interest.
