# Supplementary material for: Functional Characteristics of the Gut Microbiome in C57BL/6 Mice Differentially Susceptible to Plasmodium yoelii
Source: Front Microbiol. 2016 Sep 27;7:1520. doi: 10.3389/fmicb.2016.01520 (PMC5037233; doi:10.3389/fmicb.2016.01520)
Supplement: Supplementary file 1 [file Table_1.PDF]


Supplementary Table 1. Significantly differentially expressed mouse and bacterial genes (p ≤ 0.1), log Fold Change, and False Discovery Rate Adjusted p-values.

Source	Gene Name	Gene Product	logFC	FDR	Effect Size
Bacteria	GATM	glycine amidinotransferase	10.56203129	9.48E-07	1.3052741
	SpeA	arginine decarboxylase	7.074700747	3.60E-05	1.5428224
	NuoM	NADH-quinone oxidoreductase subunit M	6.231465975	0.000180273	1.4258402
	TusE/DsrC	tRNA 2-thiouridine synthesizing protein E	8.902038577	0.000200091	1.6800906
	PurT	phosphoribosylglycinamide formyltransferase 2	7.283113669	0.00039202	1.4756676
	DsrB	sulfite reductase beta subunit	8.385858709	0.000427852	1.6403626
	NuoN	NADH-quinone oxidoreductase subunit N	6.573771144	0.000530932	1.624988
	UreC	urease subunit alpha	4.7696686	0.000557846	1.440464
	Buk	butyrate kinase	2.102034749	0.000614343	1.6461527
	FTCD	glutamate formiminotransferase / formiminotetrahydrofolate cyclodeaminase	6.359338898	0.000614343	1.2068993
	GdhA	glutamate dehydrogenase (NADP+)	1.18441685	0.000614343	2.1202833
	NuoK	NADH-quinone oxidoreductase subunit K	7.370181564	0.002263382	1.6583615
	GlpQ/UgpQ	glycerophosphoryl diester phosphodiesterase	4.062422123	0.002263382	1.0715194
	UreA	urease subunit gamma	5.435311192	0.003406626	1.3049208
	GlcD	glycolate oxidase	-2.368672855	0.00412958	1.2855642
	RegX3	two-component system, OmpR family, response regulator RegX3	5.529495891	0.00467813	1.0360145
	NuoB	NADH-quinone oxidoreductase subunit B	3.953272974	0.005621745	1.5099251
	NuoL	NADH-quinone oxidoreductase subunit L	4.01725117	0.005621745	1.275322
	UreB	urease subunit beta	4.46892511	0.006184066	1.6861034
	IolB	5-deoxy-glucuronate isomerase	5.954879914	0.006332655	1.4514659
	FliC	flagellin	1.233262625	0.006332655	2.108153
	PTS-Aga-EIIC/AgaW	PTS system, N-acetylgalactosamine-specific IIC component	4.802058041	0.007779713	1.6118904
	SerC/PSAT1	phosphoserine aminotransferase	1.074156786	0.008473378	1.8291656
	GlpC	glycerol-3-phosphate dehydrogenase subunit C	6.354592197	0.009210915	1.5117792
	SDHA/SDH1	succinate dehydrogenase (ubiquinone) flavoprotein subunit	-4.432885653	0.009210915	0.4563344
	GctA	glutaconate CoA-transferase, subunit A	7.53547712	0.009404321	1.0240919
	NuoH	NADH-quinone oxidoreductase subunit H	4.523208595	0.013254562	1.3783323
	Ptb	phosphate butyryltransferase	2.124189103	0.016398458	1.4304567
	YgeU/XdhC	xanthine dehydrogenase iron-sulfur-binding subunit	2.154890651	0.018858598	3.3008939
	Eda	2-dehydro-3-deoxyphosphogluconate aldolase / (4S)-4-hydroxy-2-oxoglutarate aldolase	1.082844645	0.022255509	2.1391447

	E3.2.1.24	alpha-mannosidase	-1.291538009	0.025748223	2.0848625
	ALAS	5-aminolevulinate synthase	5.379671095	0.03089919	0.5861588
	DsrA	sulfite reductase alpha subunit	6.39127666	0.048137885	0.9054653
	Cgn	Cingulin	2.881220804	0.05284059	1.6751635
	MmsA/IolA/ALDH6A1	malonate-semialdehyde dehydrogenase (acetylating) / methylmalonate-semialdehyde dehydrogenase	-2.060221714	0.05284059	1.2174738
	ATPF1A/AtpA	F-type H+-transporting ATPase subunit alpha	0.844771592	0.05284059	1.3782354
	RP-S15/MRPS15/RpsO	Small Subunit Ribosomal Protein	1.062973115	0.061089357	1.6536516
	PatA	putrescine aminotransferase	2.222866162	0.061196835	1.5502705
	ITPK1	inositol-1,3,4-trisphosphate 5/6-kinase / inositol-tetrakisphosphate 1-kinase	3.690990259	0.068577927	0.6381211
	NuoA	NADH-quinone oxidoreductase subunit A	3.840319059	0.069622603	1.2586251
	LYS1	saccharopine dehydrogenase (NAD+, L-lysine forming)	1.148515411	0.072628937	1.1809167
	CheV	two-component system, chemotaxis family, response regulator CheV	1.137598186	0.072628937	1.7032707
	ABC-2.A	ABC-2 type transport system ATP-binding protein	1.268058818	0.072628937	1.1852107
	Enr	2-enoate reductase	-1.816291338	0.072628937	1.9947845
	PsaA	photosystem I P700 chlorophyll a apoprotein A1	3.908347932	0.074550244	0.8354202
	DapD	2,3,4,5-tetrahydropyridine-2-carboxylate N-succinyltransferase	1.972015719	0.076022433	1.4479565
	ALDO	fructose-bisphosphate aldolase, class I	-1.702917526	0.076022433	1.4723451
	MDH1	malate dehydrogenase	2.622145266	0.083780158	1.2696492
	RP-S6/MRPS6/RpsF	Small subunit ribosomal protein S6	0.849315223	0.08574694	2.3462309
	ATPF1B/AtpB	F-type H+-transporting ATPase subunit beta	0.738843482	0.08574694	1.3055021
	NadX	aspartate dehydrogenase	-2.056355974	0.08574694	1.9236227
	PanE/ApbA	2-dehydropantoate 2-reductase	-1.862885298	0.08574694	1.5857412
	Eno	enolase	0.677777702	0.08574694	1.0884583
	ArgG/ASS1	argininosuccinate synthase	0.921274891	0.08574694	1.163186
	FabB	3-oxoacyl-[acyl-carrier-protein] synthase I	3.561095503	0.08574694	1.1025828
	TatC	sec-independent protein translocase protein TatC	-1.55099591	0.08574694	2.0887403
	KdsA	2-dehydro-3-deoxyphosphooctonate aldolase (KDO 8-P synthase)	1.547237925	0.087896354	1.3833127
	FucK	L-fuculokinase	3.481884866	0.089880237	1.5672104
	ABC.PE.S	peptide/nickel transport system substrate-binding protein	0.835541407	0.089880237	2.2754899
	Fhs	formate--tetrahydrofolate ligase	0.703741962	0.094662643	1.4771
Mouse	Ahcyl2	adenosylhomocysteinase	6.11069988	7.54438E-05	3.0206194
	Bsg	Basigin	5.021955403	0.002347438	1.2340425

Rsrp1	Arginine/serine-rich protein 1	-3.492567218	0.002347438	1.9022358
Ndufa7	NADH dehydrogenase 1 alpha subcomplex subunit 7	3.823154723	0.015297121	3.1831989
Cyp2c55	Cytochrome P450 2C55	2.962176928	0.018513833	3.4631036
Gsdmc4	Gasdermin-C4	6.062150002	0.025095072	2.7076045
Ndufb8	NADH dehydrogenase 1 beta subcomplex subunit 8	13.81400494	0.025095072	1.1636012
Hmgcs2	Hydroxymethylglutaryl-CoA synthase	3.469436143	0.042944276	2.7083165
Rpl38	60S Ribosomal protein L38	7.647852391	0.042944276	1.5492865
Azin1	Antizyme Inhibitor 1	6.290758297	0.043120905	2.1475965
Hadhb	Trifunctional enzyme subunit beta	10.51963033	0.043120905	2.2005213
Psmb4	Proteasome Subunit beta type-4	3.343521152	0.065559612	2.54816
H3f3a	Histone H3.3	4.133121776	0.090810428	1.4220731
Lgals9	Galectin-9	5.108298738	0.090810428	2.1805035
Arpc1a	Actin-related protein 2/3 complex subunit 1A	4.444238214	0.093437914	1.5775638
Hist1h4h	Histone H4	3.780447723	0.093437914	2.4061417
Ndufs4	NADH dehydrogenase iron-sulfur protein 4	3.810497174	0.093437914	2.9942464
Pcca	Propionyl-CoA carboxylase alpha chain	6.612551547	0.093437914	1.9207781
Pnkd	Probable Hydrolase PNKD	6.633368035	0.093437914	2.6403529
Slc25a20	Mitochondrial carnitine/acylcarnitine carrier protein	-2.961285001	0.093437914	1.3733935
