# Supplementary material for: Functional Characteristics of the Gut Microbiome in C57BL/6 Mice Differentially Susceptible to Plasmodium yoelii
Source: Front Microbiol. 2016 Sep 27;7:1520. doi: 10.3389/fmicb.2016.01520 (PMC5037233; doi:10.3389/fmicb.2016.01520)
Supplement: Supplementary file 2 [file Table_2.PDF]

Supplementary Table 2. Fold Change and Cluster Analysis p-values for individual metabolites.

Compound	Pathway	Fold Change	p value	Effect Size
cysteine	Amino Acid	4.264451517	0.048392497	1.482565924
leucine/isoleucine	Amino Acid	2.436147564	0.022166626	1.743857484
aspartate	Amino Acid	2.007974347	0.009882875	2.078749142
methionine	Amino Acid	1.891779813	0.075978816	1.224121478
histidine	Amino Acid	2.261762804	0.063734379	1.305951153
phenylalanine	Amino Acid	1.64334625	0.07620887	1.22177407
tyrosine	Amino Acid	1.814098773	0.047735051	1.400186304
Xanthurenic acid	Amino Acid Metabolism	2.110636707	0.030706401	1.542142043
N-Acetyl-L-alanine	Amino Acid Metabolism	0.384640226	0.011383081	1.917705446
glycerate	Amino Acid Metabolism	6.255104016	0.011354755	2.228397124
Cysteate	Amino Acid Metabolism	2.585023342	0.079345053	1.23520779
N-acetyl-glutamate	Amino Acid Metabolism	2.81404649	0.083747638	1.2273061
uracil	Nucleotide	10.66350413	0.070807952	1.319535042
thymine	Nucleotide	7.113874218	0.085233507	1.232174327
guanine	Nucleotide	5.704544345	0.089711477	1.200954906
thymidine	Nucleotide	12.31455573	0.043687853	1.535083428
cytidine	Nucleotide	2.073567308	0.010745541	2.103744183
uridine	Nucleotide	8.48703799	0.044962788	1.533516235
dTMP	Nucleotide	2.916461634	0.050213852	1.476807495
CMP	Nucleotide	1.710297432	0.026978638	1.716741428
NADH	Nucleotide	0.341028811	0.006417009	2.121411393
adenosine	Nucleotide	2.302531269	0.061374337	1.298884329
inosine	Nucleotide Metabolism	5.002214937	0.059828075	1.392609086
hypoxanthine	Nucleotide Metabolism	5.085169781	0.027413684	1.749107264
deoxyuridine	Nucleotide Metabolism	10.38925436	0.051010272	1.472521792
xanthosine	Nucleotide Metabolism	7.301096662	0.052336967	1.459539016
dCMP	Nucleotide Metabolism	2.092157232	0.014158991	2.06543616
pyruvate	Glycolysis/TCA cycle	3.501114712	0.0031527	2.829660522
a-ketoglutarate	Glycolysis/TCA cycle	2.272752652	0.064501908	1.312544356
trehalose/sucrose	Carbon Metabolism	3.461951999	0.065543262	1.322667299
nicotinate	Vitamin	2.054801917	0.029668976	1.7110114
biotin	Vitamin	2.415017868	0.088746356	1.20510263
4-Pyridoxic acid	Vitamin Metabolism	2.554636577	0.064361789	1.356401173
Cholic acid	Bile Acid	3.786874682	0.026447811	1.694773436
Taurine	Bile Acid Biosynthesis	1.655596046	0.001840094	2.926294892
FMN	Oxydative Phosphorylation	2.718121434	0.096874767	1.17243847

sn-glycerol-3-phosphate	Glycerolipid Biosynthesis	4.255672265	0.098616854	1.166615844
1-Methyladenosine	Other	3.51953833	0.083512912	1.226670205
2-Hydroxy-2-methylbutanedioic acid	Other	3.169547335	0.068111382	1.284917215
